# Childhood Mediterranean Diet Adherence Is Associated with Lower Prevalence of Childhood Obesity, Specific Sociodemographic, and Lifestyle Factors: A Cross-Sectional Study in Pre-School Children

**DOI:** 10.3390/epidemiologia5010002

**Published:** 2023-12-23

**Authors:** Eleni Pavlidou, Sousana K. Papadopoulou, Olga Alexatou, Gavriela Voulgaridou, Maria Mentzelou, Fani Biskanaki, Evmorfia Psara, Gerasimos Tsourouflis, Nikos Lefantzis, Sophia Dimoliani, Thomas Apostolou, Anastasia Sampani, Ioanna P. Chatziprodromidou, Exakousti-Petroula Angelakou, Constantinos Giaginis

**Affiliations:** 1 Department of Food Science and Nutrition, School of the Environment, University of the Aegean, 81400 Myrina, Greece; elen.p.pavl@gmail.com (E.P.); rd.olga.alexatou@gmail.com (O.A.); maria.mentzelou@hotmail.com (M.M.); fnsd21013@fns.aegean.gr (E.P.); sdem@aegean.gr (S.D.); xeniaggelakou@hotmail.com (E.-P.A.); 2 Department of Nutritional Sciences and Dietetics, School of Health Sciences, International Hellenic University, 57400 Thessaloniki, Greece; souzpapa@gmail.com (S.K.P.); gabivoulg@gmail.com (G.V.); 3 Ministry of Education, 15180 Athens, Greece; fani.biskan@gmail.com; 4 Second Department of Propedeutic Surgery, Medical School, University of Athens, 11527 Athens, Greece; gtsourouflis@med.uoa.gr; 5 Department of Oral and Maxillofacial Surgery, Medical School, Attikon Hospital, National and Kapodistrian University of Athens, 11527 Athens, Greece; drlefa@yahoo.gr; 6 Department of Physiotherapy, School of Health Sciences, International Hellenic University, 57400 Thessaloniki, Greece; apostolouthomas@hotmail.com; 7 First Department of Pathology, Medical School, University of Athens, 11527 Athens, Greece; gerasimos.ts@gmail.com; 8 Department of Public Health, Medical School, University of Patras, 26504 Patra, Greece; ioannachatzi@med.upatras.gr

**Keywords:** Mediterranean diet, children, pre-school age, sociodemographic characteristics, anthropometric characteristics, lifestyle factors, childhood obesity, childhood asthma, diabetes mellitus type 1, quality of life

## Abstract

Background: The Mediterranean diet (MD) has been related with a decreased probability of overweight/obesity as well as central obesity at all stages of the human life, decreasing the risk of diverse disease states and improving quality of life. Over the last few years, the prevalence of childhood overweight/obesity and especially abdominal obesity has highly increased worldwide, being associated with a higher likelihood of overweight/obesity as well as central obesity at the next stages of the life during adulthood. The purpose of the present study was to explore the relationship of MD compliance with sociodemographic, anthropometry and lifestyle features in pre-school children aged 2–5 years old. Methods: This is a cross-sectional study, which includes 5188 pre-school children from diverse regions of Greece. Relevant questionnaires were applied to evaluate the sociodemographic features of the enrolled children. Anthropometric parameters were measured by relevant techniques. Qualified questionnaires were utilized for assessing several lifestyle factors such as physical activity, quality of life, breastfeeding practices, MD adherence, as well as the prevalence of childhood asthma and diabetes mellitus type I. Results: Of the enrolled children, 41.7% showed low MD compliance and 36.4% of them indicated moderated compliance, while only 21.9% of them showed a high MD adherence. Overweight/obesity was noted in 24.2% of the assigned children, while abdominal obesity was noticed in 18.2% of them. Higher MD compliance was related with an elevated prevalence of sex (boys, *p* = 0.0005), Greek nationality (*p* = 0.0088), rural type of residence (*p* = 0.0099), childhood overweight/obesity (*p* < 0.0001) and abdominal obesity (*p* < 0.0001), lower childbirth weight (*p* < 0.0001), increased physical activity (*p* = 0.0041), improved quality of life (*p* = 0.0008), exclusive breastfeeding (*p* < 0.0001), childhood asthma (*p* = 0.0001) and diabetes mellitus type 1 (*p* = 0.0002). Conclusions: A higher MD adherence is associated with specific sociodemographic, better anthropometric, and beneficial lifestyle factors in pre-school children. However, MD compliance remains low or moderate in the vast majority of children aged 2–5 years old. Thus, future public strategies and policies should be performed to inform parents of the potential beneficial effects of MD against obesity and related chronic diseases at the next stage of their children’s lives.

## 1. Introduction

The Mediterranean diet (MD) is considered the most studied dietary pattern, which can exert multiple beneficial effects as a preventive and/or co-treatment factor against several chronic human diseases in all age groups, including pre-school children [1,2]. The main characteristic of the MD is that it includes a plethora of healthy foodstuffs such as fruits, vegetables, nuts, cereals, whole grains, and extra virgin oil, and numerous bioactive compounds, such as ω-3 fatty acids, flavonoids, and polyphenols, which can exert antioxidant, anti-inflammatory, anti-thrombotic, anti-cancer, anti-neurodegenerative and anti-aging properties [3,4,5]. Moreover, there is currently substantial evidence that MD-related nutritional interventions exert a considerable impact on decreasing the body mass index (BMI), and lowering obesity in both children and adolescents, and especially in those with pre-existing overweight or obesity [6]. Notably, a high MD adherence can reduce the risk of diverse obesity-related disorders, including hypertension, dyslipidemia, diabetes mellitus type 2, cardiovascular diseases, and certain types of cancer [7]. Interestingly, several medicinal plants and herbs of the MD include various phytochemicals with anti-obesity properties [8,9]. More to the point, the MD constitutes a conglomeration of phytochemicals like resveratrol, berberine, carvacrol, curcumin, capsaicin, quercetin, luteolin, thymol, etc., which can act in opposition to obesity and metabolic diseases [9,10,11]. The mechanisms of their anti-obesity activities include the suppression of adipocyte differentiation, browning of the white adipose tissue, inhibition of enzymes like lipase and amylase, inhibition of inflammatory conditions, enhancement of the intestinal microbiome, and decrease in obesity-related genes’ expression [9,10,11].

Overweight and obesity in childhood and adolescence have been recognized as serious public health concerns nowadays because of its epidemic widespread and the associated considerable morbidity and mortality, as well as the rising public healthcare expenses [12,13]. The incidence of overweight and obesity in the childhood population has considerably increased in the last 3–4 decades worldwide, increasing from 4% in 1975 to more than 18% in 2016 [14]. Remarkably, children affected by obesity are characterized by a five-fold elevated probability of presenting persistent obesity in adulthood [15,16,17]. Especially, childhood obesity and its comorbidities constitute crucial risk factors for several pathological conditions, comprising three of the major non-communicable diseases globally: diabetes mellitus type 2, cardiovascular disease, and cancer [15,16,17]. The most remarkable characteristic of childhood obesity is related to the capability of adipose tissue to store lipids, being increased in size during excessive calorie consumption [18]. Cellular and lipid turnovers influence adipose tissue volume and are directly associated with metabolism health [19]. However, the molecular mechanisms by which adipose tissue is increased and whether this influences systemic metabolism homeostasis remain yet unknown. In addition, the potential molecular mechanisms by which enhanced adiposity expands from childhood to adulthood and its subsequent involvement in metabolic status remain still unidentified [18,19].

Several perinatal, sociodemographic and lifestyle factors may also increase the prevalence of childhood obesity. Higher maternal pre-pregnancy BMI directly correlates with an increased risk of childhood obesity [20,21]. Higher childbirth weight may also enhance the risk of childhood obesity [21,22]. Maternal excessive gestational weight gain (GWG) may lead to fetus macrosomia and high childbirth weight that could in turn increase the risk of childhood obesity [23,24]. Moreover, gestational diabetes mellitus has been found to double the probability of childhood overweight/obesity at the ages of 2–5 years old, regardless of diverse confounders [25,26]. Childhood obesity has also been affected by socioeconomic factors, including low financial status, low parental education, decreased fruits’ intake, low sleep duration, and parental obesity [27,28]. In the last few years, the COVID-19 quarantine could also exert a deleterious impact in the worldwide attempts to reduce childhood obesity [29,30]. School closures, alteration in daily activities, and the loss of structure and control were adversely related with children obesity concerning the COVID-19 confinement period [29,30]. There is also substantial evidence that physical activity between childbirth and the age of 5 years old may further prevent obesity both in the short and long-term [31,32].

A common risk factor related with children obesity pertains to nutrition or the specific kind of dietary habits that the children implement in their daily life [33]. A recent systematic review, including 28 clinical studies, assessed both nutritional and physical activity interventional approaches intended at decreasing childhood obesity, from birth to 5 years old [34]. This review showed that 13 (46%) interventional studies resulted in better children obesity indicators like body mass index (BMI) z-score and body fat proportion, 12 of which contained both parental/family-based interventional approaches in combination with adapting healthier children’s dietary habits and physical activity behaviors [34]. Several reviews have also analyzed the dietary interventional approaches, which can be applied to prevent children’s excessive body weight [35,36,37]. The promotion of breastfeeding, the protein content decrease in formulated milks, and nutritional habits at the initial 12–24 months, including family and schools in interventional approaches which encourage physical activity and healthy dietary habits, have been considered as promising strategies in the reduction of the risk of obesity [35,36,37]. In this aspect, a multidimensional approach that takes into consideration diverse areas of intervention, has been supported to be more effective in preventing childhood obesity [37,38]. Importantly, combined strategies, including several components such as nutrition and physical activity at first, at different levels (individual, family, school, and institutional) are considered essential for the prevention of childhood obesity [37,38]. However, in a literature review, the majority of the interventional surveys did not lead to reliable impacts on altering the children’s BMI status [39]. The above surveys merely found minor body weight decreases, clinically inappropriate, or no impacts at all [39]. Moreover, a systematic review of meta-analyses has shown that there are discrepancies between meta-analyses on observational and interventional studies [40]. In this systematic review, combined interventions, containing physical activity and dietary modifications, were found to represent the most efficient means for tackling childhood obesity [40]. However, the authors strongly recommended the demand for further research about the most effective multidimensional prevention strategy [40].

In Europe, the highest incidence of childhood overweight and obesity was recorded in the countries of the Mediterranean Basin such as Greece, Cyprus, Italy, and Spain, where about 25% of pre-school children were classified as overweight or obese [41]. The higher prevalence estimates were in Italy (32.4%), Greece (29.6%), and Portugal (26.4%) [41]. During the initial 1000 days, the fetus and infant are exposed to risk factors, which affect their growth, development, and future health state, being underlined as a critical period for the expansion of obesity [42]. In Greece, several studies have been performed to evaluate the prevalence of overweight and obesity in children and the associated risk factors. However, almost all of them have been focused on children who were older than 6 years and not on pre-school children at the age of 2–5 years. In this context, the current study aims to evaluate the incidence of overweight and obesity in pre-school children, highlighting the potential association of MD adherence with childhood overweight/obesity, taking into consideration diverse sociodemographic and lifestyle factors that may be involved in the excessive body weight of this age group of children.

## 2. Materials and Methods

### 2.1. Study Population

**Figure 1:**
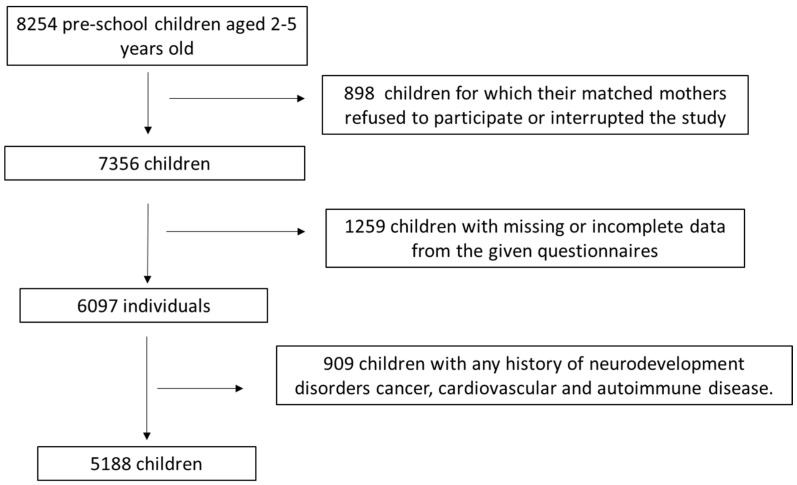
Flow chart diagram for the enrolled study population.

This is a cross-sectional study in which 8254 pre-school children aged 2–5 years old and their matched mothers were initially enrolled from 10 geographically diverse Greek regions, namely Athens, Thessaloniki, Larissa, Patra, Alexandroupolis, Kalamata, Ioannina, Crete and South and North Aegean. Recruitment to the study occurred between May 2016 and September 2020. The inclusion criteria for the initial enrollment were children aged 2–5 years old whose mothers had a singleton childbirth before 2–5 years. All participating children were disease-free despite the possible development of asthma or diabetes mellitus I at the first stages of their life. Among the 8254 firstly assigned children and their paired mothers, 898 (10.9%) of the mothers either refused to participate at the beginning of the study or interrupted their participation in the study during the completion of the given questionnaires. Among the remaining 7356 assigned children and their paired mothers, 1259 of them (17.1%) were not included in the survey because of missing or not complete data. Amongst the remaining 6097 children and their paired mothers, 909 (14.9%) of the participating children were afterward excluded from the survey because of any history of childhood neurodevelopment diseases (e.g., autism spectrum disease, attention deficit hyperactivity disease, mental retardation, motor disease) as well as cancer, cardiovascular and autoimmune disorders. The presence of any of the above diseases were self-reported by the enrolled mothers. A total of 5188 children and their paired mothers were finally included in the study analysis after applying the above inclusion and exclusion criteria, leading to a final response rate of 62.9%. A flow chart diagram of the study enrollment is presented in Figure 1.

All participants’ data remained strictly private. The enrolled mothers of the children were informed regarding the aim of the survey and signed a consent form, giving their approval to publish their personal information namelessly. The survey was approved by the Ethical Agency of the University of the Aegean (ethical approved protocol: no 12/14.5.2016) and was in accordance with the World Health Organization (52nd WMA General Assembly, Edinburgh, Scotland, 2000). Sample size estimation was determined using the PS: Power and Sample Size calculator program. The randomization was performed utilizing a sequence of random binary numbers (i.e., 001110110 in which 0 signified assignment and 1 no assignment to the survey). The calculation of the power of the study sample size has indicated a power of 88.4%.

### 2.2. Study Design

At the time of study, relevant questionnaires were applied to assess the sociodemographic features of the enrolled children such as child age, sex (boys vs. girls), nationality (Greek vs. other), and type of residence (urban vs. rural) through one-to-one interviews among their assigned matched mothers and qualified nutritionists or dietitians to reduce recall biases. Child anthropometric parameters such as body weight, waist circumference and height were measured at the time of study by qualified personnel. Body weight was measured utilizing the same electronic scale, and height was determined by a portable stadiometer [23,43,44]. The weight was measured almost to the closest 100 g, and the height was determined to the closest 0.50 cm. The International Obesity Task Force (IOTF) reference was used to classify pre-school children as normal weight, overweight or obese [45,46]. The waist circumference was determined at the midpoint between the lower margin of the last palpable ribs and the top of the iliac crest [47]. Waist circumference (WC) constitutes a simple indicator, which is one of the criteria for the metabolic syndrome and has been well-established as a useful risk index of abdominal obesity, independent of measuring BMI [48,49]. According to the Third National Health and Nutrition Examination Survey and several substantial studies, WC has used as an indicator of abdominal obesity. In fact, for boys aged 2 to 5 years, the 90th percentile (58.25 cm; sensitivity, 48.0%; specificity, 91.5%), while for girls aged 2 to 5 years, the 62nd percentile (53.27 cm; sensitivity, 71.4%; specificity, 63.1%), were used to classify children as presenting or not presenting abdominal obesity [50,51].

The Waist to Heigh (WtHR) was also estimated by dividing waist measurement by height measurement. In this aspect, it should be noted that WtHR has been considered superior to BMI [50,51]. In fact, it has been identified as an effective index of central obesity that has been identified as an efficient anthropometry parameter for estimating more effectively the risk of several cardiometabolic diseases such as diabetes mellitus 2, myocardial infraction, and stroke [50,51]. WtHR was determined by dividing WC by height, and 0.50 was used as a cutoff for identifying abdominal obesity [50,51]. Childbirth weight was also retrieved by the mothers’ medical records and was categorized as low (<2500 gr), normal (2500–4000 gr) and high (>4000 gr) as suggested by the majority of the literature [52].

The Preschool-age Children’s Physical Activity Questionnaire (Pre-PAQ, see Supplementary File S1) was applied to assess the activity levels of preschool-age children in the home environment [53]. Pre-PAQ is a promising questionnaire with high reliability and validity which can be completed by the parents and can be used to assess activity behavior in large-scale population surveys conducted on preschool-age children [53]. Based on the Pre-PAQ, the enrolled children were classified into three categories: (a) low, (b) medium and (c) high physical activity levels. The Pre-PAQ has the advantage that it can provide condensed data on the nature, level, and duration of a child’s activity behavior. The Pre-PAQ can also simultaneously determine parental, family and neighborhood factors, which could affect child’s behaviors [53].

The Pediatric Quality of Life Inventory (PedsQL) was applied to assess the health-related quality of life in pre-school children. This is a brief measure which can efficiently be completed by parents [54]. The PedsQL inventory takes around 5 min to be completed and can be self-administered by parents who had a child aged 2–5 years old after being analytically informed by a trained administrator [54]. The PedsQL constitutes a 23-item generic health state measurement of high reliability and validity. More to the point, parents’ and children’ forms evaluate 5 domains of health (physical functioning, emotional functioning, psychosocial functioning, social functioning, and school functioning) in children and adolescents of ages from 2 to 18 years old [54,55]. The PedsQL questionnaire (See Supplementary File S2) can distinguish between healthy children from those with chronic pathological states, while it can also be associated with indicators of health care access, days missed from school, days sick in bed or too ill to play, and days requiring care [54,55].

Childhood MD compliance of the enrolled pre-school children was assessed using the KIDMED questionnaire [56]. The KIDMED questionnaire (see Supplementary File S3) is one of the most widely used scoring systems to assess adherence to the MD. This tool has high reliability and validity and contains 16 questions for assessing children’s nutritional habits. Every question has a “yes” or “no” response which varies between −1 (negative connotation) and +1 (positive connotation). Twelve questions take a positive score and four questions take a negative score. More to the point, it includes 4 questions denoting a negative connotation to the MD (consumption of fast food, baked goods, sweets, and skipping breakfast) and 12 questions denoting a positive connotation (consumption of oil, fish, fruits, vegetables, cereals, nuts, pulses, pasta or rice, dairy products, and yoghurt). Total KIDMED scores range from 0 to 12 and are classified as follows: ≥8 points, good; 4–7 points, average; and ≤3 points, poor MD adherence [56].

Mothers were asked whether they were exclusively breastfeeding for at least 4 months. To overcome recall biases, the mothers were asked about exclusive breastfeeding for at least four months because at the end of the 4th month and the beginning of the 5th month most of them were counselled to slowly include pulp foods to the feeding practices of their children and thus they can remember more exactly this time point, making their responses more consistent. Mothers breastfeeding for smaller intervals were not capable of answering with absolute certainty concerning the exact breastfeeding interval [25,26]. Childhood asthma was diagnosed by certified clinicians based on the International Study of Asthma and Allergies in [57,58]. Children diagnosed with diabetes mellitus type I data were self-reported by their matched mothers.

Clarifying detailed advice was systematically given to the mothers of the enrolled children by trained dietitians and nutritionists concerning the accomplishment of the questionnaires, and a comprehensive demonstration of the questions to obtain reliable responses was applied to increase the validity of the mothers’ responses. All questionnaires were answered by the mothers of the children under study.

### 2.3. Statistical Analysis

Student’s *t*-test was applied for the continuous variables that were characterized by normal distribution. The Kolmogorov–Smirnov test was used to evaluate if every continuous variable was characterized by normal distribution. Chi-square was applied for categorical variables. The quantitative variables, which followed normal distribution, were expressed by mean value ± standard deviation (SD). Non-parametric analysis applying the Mann–Whitney test for the variables which did not follow normal distribution was used. The quantitative continuous variables, which did not follow normal distribution, were expressed by median value (Interquartile Range, IQR). The qualitative variables were stated as absolute or relative frequencies. To assess if MD could independently be associated with sociodemographic, anthropometric, and lifestyle factors, multivariate binary logistic regression analysis was applied by adjusting for possible confounders. The Statistica 10.0 software was applied for the statistical analysis of the data of the present study (Informer Technologies, Inc., Hamburg, Germany).

## 3. Results

### 3.1. Descriptive Statistics of the Study Population

This cross-sectional study included 5188 pre-school children aged 2–5 years old. All the descriptive statistics are included in Table 1. The mean age of the enrolled children was 4.1 ± 1.5 years old. Of the children, 49.1% were boys and 50.9% were girls. The vast majority (92.5%) of the assigned children were Greeks, while 4.4% of them had different nationalities. Of the enrolled children, 65.5% lived in urban regions in Greece and the remaining 34.5% lived in rural regions.

As far as the anthropometrics characteristics are concerned, based on BMI, 17.0% of the assigned children were affected by overweight and 7.2% by obesity, and thus 24.2% were overweight/obese overall. Based on WtHR, 18.2% of the participating children showed abdominal obesity. According to WC, 21.4% of the enrolled children showed abdominal obesity. Based on childbirth weight, 7.0% of the assigned children had high birth weight, while 8.4% had low birth weight.

Concerning physical activity levels according to Pre-PAQ, 45.6% of the children had low and 41.8% had medium physical activity levels and only 12.6% of the enrolled children showed high physical activity levels. The mean quality of life score according to the PedsQL questionnaire was 68.5 ± 7.1. Moreover, 7.4% of the enrolled children were diagnosed with asthma and 6.5% of them were diagnosed with diabetes mellitus type 1. Of the enrolled children, 50.9% were breastfed exclusively for at least 4 months. Based on the KIDMED questionnaire, only 21.9% of the assigned study showed high MD adherence, while 36.4% of them had moderate MD compliance and 41.7% of them exhibited low MD adherence.

### 3.2. Association of Children MD Adherence with Sociodemographic and Anthropometric Characteristics

Boys had significantly higher levels of MD adherence compared to girls (Table 2, *p* = 0.0005). Greek children showed higher MD compliance compared to children of other nationalities (Table 2, *p* = 0.0088). Children living in rural regions showed greater levels of MD adherence compared to children living in urban regions (Table 2, *p* = 0.0099). Children which were classified as overweight, or obese, exhibited considerably decreased levels of MD adherence compared to normal weight children (Table 2, *p* < 0.0001). Abdominal obesity classified either by WtHR or WC was significantly more frequently observed in children with lower levels of MD compliance (Table 2, *p* < 0.0001).

### 3.3. Association of Childhood MD Adherence with Lifestyle Factors, Childhood Asthma and Diabetes Mellitus 1

Higher levels of MD adherence were significantly more frequently observed in children with greater physical activity levels and higher quality of life scores (Table 2, *p* = 0.0041 and *p* = 0.0008, respectively). Exclusive breastfeeding was considerably more frequently noted in children with higher MD compliance compared to those that were not breastfed at all or breastfed for less than 4 months (Table 2, *p* < 0.0001). Children diagnosed with asthma or diabetes mellitus 1 showed significantly lower levels of MD adherence than those without asthma or diabetes mellitus 1 (Table 2, *p* = 0.0001, and *p* = 0.0002, respectively).

### 3.4. Multivariate Binary Logistic Regression Analysis for MD Adherence of the Study Population

In multivariate binary logistic regression analysis, childhood MD adherence was independently associated with anthropometric factors such as BMI, WtHR and WC, as well as with physical activity, quality of life, breastfeeding practices, childhood asthma and diabetes mellitus type 1 (*p* < 0.05). Specifically, children with low or moderate MD compliance showed a more than two-fold greater prevalence of overweight or obesity compared to children with elevated MD compliance (Table 3, *p* = 0.0024). Children presenting low or moderate levels of MD compliance also had about two-fold higher incidence of abdominal obesity than those with high MD adherence for both WtHR and WC indices (Table 3, *p* = 0.0021 and *p* = 0.0038, respectively).

Children with low or moderate levels of MD compliance exhibited a 57% higher prevalence of low physical activity levels compared to those with increased MD compliance (Table 3, *p* = 0.0175). Children presenting low or moderate levels of MD compliance had a more than two-fold prevalence of poor quality of life compared to children with high MD compliance (Table 3, *p* = 0.0086). A more than two-fold prevalence of no breastfeeding was observed in children with low or moderate MD compliance compared to those with high MD compliance (Table 3, *p* = 0.0056). Children with low or moderate levels of MD adherence exhibited a 69% greater incidence of asthma compared to children with high MD adherence (Table 3, *p* = 0.0182). Accordingly, children with low or moderate levels of MD adherence showed a 48% higher prevalence of diabetes mellitus type 1 than children presenting high MD adherence (Table 3, *p* = 0.0289).

## 4. Discussion

This is one of the few studies in Greece that has assessed the prevalence of overweight and obesity in pre-preschool children aged 5–2 years old and the first study that has evaluated the association of MD adherence with diverse sociodemographic, anthropometric and lifestyle factors in this age group. This cross-sectional study conducted on an adequate child population has found that higher MD adherence was independently associated with a lower prevalence of childhood overweight and obesity as well as with a lower incidence of abdominal obesity. In addition, higher levels of MD compliance were independently related with greater physical activity, better quality of life, exclusive breastfeeding as well as a lower prevalence of childhood asthma and diabetes mellitus type 1 in pre-school age. Moreover, higher levels of MD adherence were significantly more frequently observed in children with Greek nationality, who lived in rural regions, and who had a normal birth weight. However, these associations were considerably attenuated and became non-significant after adjusting for diverse confounders.

In agreement with our results, a previous study in our country has shown that lower KIDMED scores were related with an increased risk of childhood overweight/obesity and especially in children living in nuclear families [59]. Another study conducted on primary-school Greek children found a prevalence of 26% of childhood overweight/obesity, which is close to our findings [60]. In this study, children’s compliance to the MD was inversely related with body weight, either parents were away from or close to this nutritional pattern [60]. However, these studies were performed in primary-school Greek students and did not include pre-school children. A nationwide survey in Greece also found a high incidence of overweight/obesity in children aged 10–12 years old [61]. In line with our findings, this substantial study revealed that children from semi-urban or rural regions exhibited greater KIDMED scores. In addition, children with greater KIDMED scores adopted healthier dietary habits and presented greater physical activity in agreement with our findings [61]. A national cross-sectional study among 1140 children aged 9–13 years old in Cyprus also found that higher MD compliance was inversely related with childhood obesity [62]. However, this survey highlighted that other factors, and especially physical activity, the mother’s obesity, dietary beliefs, and attitudes may exert a more considerable effect than MD adherence for controlling childhood obesity [62].

A cross-sectional survey performed on 2049 pre-school children aged 3–6 years has recently been carried out in Taizhou of China [63]. This study has shown that pre-school children with low scores of satiety responsiveness and slowness in eating (‘Food Avoidant’ subscales) and greater scores in enjoyment of food (‘Food Approach’ subscales) had a higher possibility of being affected by obesity [63]. A previous study compared three surveys from different parts of Germany, assessing the prevalence and remission of overweight and obesity at the age of 2 years old until school admission and from school admission to fourth grade [64]. At the pre-school age, the pooled incidence of overweight was 8.2% compared with a remission rate of 62.6%, yielding a prevalence at school entry of 10.7%. At the primary school age, the pooled prevalence of overweight reached to 14.6%, whereas the remission rate was reduced to 17.7%, yielding a prevalence of 23.7% in fourth grade. An analogous pattern was also noted for obesity [64]. A study performed in pre-school children in Kenya reported that approximately 18% of the enrolled children were overweight and 4% were obese, which is in accordance with our finding [65]. Accordingly, this study revealed a significant association between large childbirth weight and childhood overweight/obesity [65].

In agreement with our study, the ToyBox-study has indicated that the proportion of pre-school children having a low Diet Quality Index (DQI), not adapting to both step and screen time (ST) references was very high, and it was related with an increased likelihood of being affected by overweight or obesity [66]. A cross-sectional study evaluating data from the National Health and Nutrition Examination Survey (NHANES) has shown that greater MD adherence was significantly related with a lower risk of childhood overweight and obesity; however, the enrolled children had a broad range of ages from 2 to 11 years old [67]. Moreover, in a Spanish population of pre-school children aged 4–5 years old, a higher adherence of children to the MD was independently associated with breastfeeding for at least 6 months compared to their peers who were never breastfed [68]. Accordingly, in a randomized, parallel trial of children aged 3–5 years old, high MD adherence resulted in a reduction in BMI after one year and at the end of the follow-up interval [69]. A cross-sectional study including 982 schoolchildren at the age of 4–6 years from Chile, Colombia, and Spain has also documented that a better lifestyle, including higher MD adherence, was significantly associated with a lower risk of abdominal obesity [70].

Furthermore, a cross-sectional study conducted on 969 Chilean and Colombian schoolchildren aged about 5 years old has shown that abdominal obesity and excess body weight were associated with an unhealthy lifestyle, including lower levels of MD adherence [71]. In line with our findings, a cross-sectional, observational study in Croatia indicated that almost half (49%) of the participating pre-school children exhibited decreased KIDMED scores, revealing reduced MD compliance, 37% exhibited moderate KIDMED scores, whereas merely 14% showed increased MD adherence [72]. Accordingly, KIDMED scores were observed optimal in 34.1%, moderate in 57.0%, and very low in 8.9% in Turkish pre-school children [73]. A population-based Norwegian study conducted on 34,074 pre-school children demonstrated that a high diet quality index related with MD adherence in early childhood could decrease the probability of overweight in later childhood [74]. Moreover, a small breastfeeding period, lower physical activity, and short sleep duration and long screentime at 18 months were related with a 2–3% lesser diet quality index in children aged 3 years old [74]. A substantial cohort study also showed that greater MD compliance in children aged 4 years old was related with a decreased likelihood of developing overweight, obesity, as well as central obesity at the age of 8 years old [75].

A national survey including 174,209 students at the age of 6–18 years from several geographical regions of Greece (EYZHN study) clearly demonstrated that greater adherence to the Mediterranean lifestyle was significantly related with a decreased risk of being overweight, obesity as well as central obesity [76]. In this study, the MediLIFE-index was used as indicator of Mediterranean lifestyle, which simultaneously includes MD adherence, physical activity levels, sedentary behavior, and sleep duration [76]. This study highlighted that an overall healthy lifestyle may prevent childhood and adolescent obesity, even if it was not limited to the pre-school age [76]. A Spanish study conducted on 619 children who were on average 4.7 years old supported evidence that higher MD adherence and greater cardiorespiratory fitness were associated with a lower waist circumference in pre-school children [77]. This study pointed MD adherence and cardiorespiratory fitness as effective modifiable factors to be targeted by educational approaches promoting the prevention of abdominal obesity and subsequent obesity-associated comorbidities [77]. A systematic review critically analyzed data concerning the nutritional habits of pre-school children living in the Mediterranean countries of the European Union [78]. This study revealed that young children consumed fruit and vegetables quite frequently; however, they also consumed sugared beverages and snacks in most of the countries. Elevated energy and protein consumptions primarily from dairy products were also found in most of the countries. Notably, an increased prevalence of overweight and obesity was observed at the first age of the children’s life [78]. Moreover, both early intake of energy-dense foods and overweight were noted to follow across toddler and pre-school ages. Most of the above enrolled children living in these countries exhibited a decreased compliance of a Mediterranean-like diet that was subsequently related with an increased risk of overweight and obesity [78].

In agreement with our findings, a Spanish prospective study conducted on 50 girls and 54 boys aged 1–5 years showed that a higher adherence of a traditional MD may considerably contribute to the attenuation of symptom severity of children who suffered from asthma [79]. Moreover, another Spanish study in 1000 pre-school children aged 1.5–4 years old found that a decreased fruit intake and increased meat consumption had a negative effect on wheezing, rhinitis, or dermatitis [80]. A recent systematic review including 12 studies also reported that the MD may exert a protective role against childhood asthma [81]. However, the authors have emphasized that the currently available studies suffered from high heterogeneity and several limitations, highlighting the need for well-designed randomized controlled trials, which should be focused to children’s populations to establish more robust findings [81]. Moreover, we found that increased MD compliance was related with decreased incidence of diabetes type 1 in pre-school children. However, there is not any survey so far examining the effect of MD adherence in the prevalence of diabetes type 1 in this age group. There is only a recent cross-sectional study conducted in children with an average age of 11.4 years [82]. This study suggested that MD adherence could improve glycemic control in children, and this issue should be taken into consideration during the nutritional education of children with diabetes type 1 and their families [82].

Our study exhibits several strengths as it was performed on a sample size adequate enough of 5188 pre-school children assigned from various regions of our country. In addition, our research is one of the few studies evaluating the potential impacts of MD compliance in the prevalence of overweight/obesity in pre-school age, taking into consideration diverse sociodemographic and lifestyle factors. Another strength of our research is that one-to-one interviews among the enrolled matched mothers and the trained staff were conducted to reduce recall biases. The thorough explaining information as well as the detailed presentation of the questionnaires that were performed in the one-to-one interviews could additionally minimize possible recall biases, and also enhance the validity and the consistency of the mothers’ responses. Moreover, anthropometric parameters were measured by qualified personnel, and thus they were not self-reported. In addition, we used validated questionnaires to assess MD adherence, quality of life, and physical activity of the enrolled children, while childhood asthma was diagnosed by specialized physicians. Lastly, in our study, the assessment of childhood overweigh/obesity was not restricted to BMI classification, but it was expanded to the evaluation of WC and WtHR classification, which are related with abdominal obesity, that in turn are more effective factors for the estimation of the probability of developing chronic diseases at the next stages of the children’s lives.

Our study also has certain limitations. The cross-sectional design of our study decreases the probability of providing conclusive findings. Hence, no highly reliable conclusions about causality may be extracted due to our study design. Potential risks of recall biases, and especially regarding the self-reported questions, even if we have applied well-organized one-to-one interviews, may exert confounding effects. Nonetheless, self-reported data have comprehensively been used in epidemiological surveys, having high reliability and accuracy to estimate diverse outcomes. Moreover, it remains still the probability of unmeasured confounders like several aspects of mental health, sleep disturbances, and the presence of eating disorders of the assigned children, despite our systematic approaches to confounders’ adjustment. Thus, it is still possible that residual confounding could affect our findings even if we have applied a thorough adjustment for multiple confounding factors. Moreover, the questionnaires used in our study were compiled by the mothers of the children under study, which may induce a consistent bias as the parents, and especially the mothers, tend to give a better picture of their children’s eating habits. In addition, children 2–5 years old are highly influenced in their behavior by the family’s eating habits that, in turn, can be modulated by socioeconomic and cultural factors. The above may imply that the children’s eating habits may reflect the family’s diet. As children and adolescent eating patterns have changed over the last few years, a new version of the questionnaire determining MD adherence in children and adolescents, KIDMED 2.0, with good reliability and validity was developed after the performance of the present study [83]. In this aspect, it would be interesting to verify our findings with the new KIDMED 2.0 questionnaire in the future.

## 5. Conclusions

This is one of the few currently available studies highlighting that a higher MD adherence may be associated with lower prevalence of overweight/obesity and abdominal obesity in pre-school age, being also associated with several sociodemographic and lifestyle factors. The high prevalence of overweight/obesity and most importantly of abdominal obesity in pre-school age reinforces the urgent need for the development and implementation of well-organized public strategies and policies that could inform future mothers about the beneficial effects of MD adherence at the early stages of their children’s lives in conjunction with other lifestyle factors, e.g., physical activity against childhood overweight and obesity. Nutritional counselling and support for future mothers is strongly recommended to confront the epidemic of childhood obesity and their related complications.

## Figures and Tables

**Table 1:** Descriptive statistics of the enrolled children.

Characteristics (*n* = 5188)	Descriptive Statistics
**Age (mean ± SD; years)**	4.1 ± 1.5
**Sex (*n*, %)**	
Boys	2546 (49.1%)
Girls	2642 (50.9%)
**Nationality (*n*, %)**	
Greek	4938 (95.2%)
Other	250 (4.8%)
**Type of residence (*n*, %)**	
Urban	3399 (65.5%)
Rural	1789 (34.5%)
**BMI (*n*, %)**	
Normal weight	3934 (75.8%)
Overweight	880 (17.0%)
Obese	374 (7.2%)
**Abdominal obesity status (WtHR) (*n*, %)**	
No	4244 (81.8%)
Yes	944 (18.2%)
**Abdominal obesity status (WC) (*n*, %)**	
No	4076 (78.6%)
Yes	1112 (21.4%)
**Birth weight (*n*, %)**	
Low child weight (<2500 gr)	436 (8.4%)
Normal child weight (2500–4000 gr)	4391 (84.6%)
High child weight (>4000 gr)	361 (7.0%)
**Physical activity ^1^ (*n*, %)**	
Low	2368 (45.6%)
Medium	2169 (41.8%)
High	651 (12.6%)
**Quality of life ^2^ (mean score ± SD)**	68.5 ± 7.1
**Exclusive breastfeeding (*n*, %)**	
No	2549 (49.1%)
Yes	2639 (50.9%)
**Asthma (*n*, %)**	
No	4795 (92.4%)
Yes	393 (7.6%)
**Diabetes type 1 (*n*, %)**	
No	4851 (93.5%)
Yes	337 (6.5%)
**MD adherence ^3^ (*n*, %)**	
Low	2164 (41.7%)
Moderate	1890 (36.4%)
High	1134 (21.9%)

^1^ Physical activity was assessed by the Preschool-age Children’s Physical Activity Questionnaire (Pre-PAQ) [53]. ^2^ Quality of life was assessed by the Pediatric Quality of Life Inventory (PedsQL) questionnaire [54]. ^3^ Mediterranean diet (MD) adherence was assessed by the KIDMED (Mediterranean Diet Quality Index for children and adolescents) questionnaire [55].

**Table 2:** Associations of Mediterranean diet compliance (KIDMED) with sociodemographic, anthropometry and lifestyle factors of the enrolled pre-school children.

Characteristics (*n* = 5188)	Mediterranean Diet Adherence ^1^			
	Low 2164 (41.7%)	Moderate 1890 (36.4%)	High 1134 (21.9%)	*p*-Value
**Age (mean ± SD; years)**	7.61 ± 1.06	7.58 ± 1.09	7.56 ± 1.08	*p* = 0.2391
**Sex (*n*, %)**				*p* = 0.0005
Boys	986 (45.6%)	958 (50.7%)	602 (53.1%)	
Girls	1178 (54.4%)	932 (49.3%)	532 (46.9%)	
**Nationality (*n*, %)**				*p* = 0.0088
Greek	2038 (94.2%)	1806 (95.6%)	1094 (96.5%)	
Other	126 (5.8%)	84 (4.4%)	40 (3.5%)	
**Type of residence (*n*, %)**				*p* = 0.0099
Urban	1478 (68.3%)	1214 (64.2%)	707 (62.3%)	
Rural	686 (31.7%)	676 (35.8%)	427 (37.7%)	
**BMI (*n*, %)**				*p* < 0.0001
Normal weight	1205 (55.7%)	1677 (88.7%)	1052 (92.8%)	
Overweight	669 (30.9%)	148 (7.8%)	63 (5.5%)	
Obese	290 (13.4%)	65 (3.5%)	19 (1.7%)	
**Abdominal obesity (WtHR) (*n*, %)**				*p* < 0.0001
No	1547 (71.5%)	1657 (87.7%)	1040 (91.7%)	
Yes	617 (28.5%)	233 (12.3%)	94 (8.3%)	
**Abdominal obesity (WC) (*n*, %)**				*p* < 0.0001
No	1466 (67.7%)	1598 (84.5%)	1012 (89.2%)	
Yes	698 (32.3%)	292 (15.5%)	122 (10.8%)	
**Birth weight (*n*, %)**				*p* < 0.0001
Low (<2500 gr)	212 (9.8%)	159 (8.4%)	110 (9.7%)	
Normal (2500–4000 gr)	1655 (76.5%)	1629 (86.2%)	971 (85.6%)	
High (>4000 gr)	297 (13.7%)	102 (5.4%)	53 (4.7%)	
**Physical activity ^2^ (*n*, %)**				*p* = 0.0041
Low	1003 (46.4%)	870 (46.0%)	495 (43.6%)	
Medium	918 (42.4%)	792 (41.9%)	459 (40.5%)	
High	243 (11.2%)	228 (12.1%)	180 (15.9%)	
**Quality of life ^3^ (mean score ± SD)**	61 ± 6.8	67.9 ± 7.2	73.4 ± 7.5	*p* = 0.0008
**Exclusive breastfeeding (*n*, %)**				*p* < 0.0001
No	1156 (53.4%)	882 (46.7%)	511 (45.1%)	
Yes	1008 (46.6%)	1008 (53.3%)	623 (54.9%)	
**Asthma (*n*, %)**				*p* = 0.0001
No	1942 (89.7%)	1776 (94.0%)	1077 (95.0%)	
Yes	222 (10.3%)	114 (6.0%)	57 (5.0%)	
**Diabetes type 1 (*n*, %)**				*p* = 0.0002
No	1991 (92.0%)	1776 (94.0%)	1084 (95.6%)	
Yes	173 (8.0%)	114 (6.0%)	50 (4.4%)	

^1^ Mediterranean diet (MD) adherence was assessed by the KIDMED (Mediterranean Diet Quality Index for children and adolescents) questionnaire [55]. ^2^ Physical activity was assessed by the Preschool-age Children’s Physical Activity Questionnaire (Pre-PAQ) [53]. ^3^ Quality of life was assessed by the Pediatric Quality of Life Inventory (PedsQL) questionnaire [54].

**Table 3:** Multivariate logistic regression analysis for Mediterranean diet compliance of pre-school children aged 2–5 years old.

Characteristics	Mediterranean Diet Adherence ^1^ (Low vs. Moderate & High)	
	OR * (95% CI **)	*p*-Value
**Age** (Over/Below mean value)	0.98 (0.23–1.74)	*p* = 0.57483
**Gender** (Male/Female)	1.27 (0.64–1.91)	*p* = 0.1728
**Nationality** (Greek/Other)	1.18 (0.61–1.86)	*p* = 0.2011
**Type of residence** (Rural/Urban)	1.32 (0.68–1.97)	*p* = 0.2879
**BMI** (Overweight & Obese/Normal weight)	2.27 (1.98–2.59)	*p* = 0.0024
**Abdominal obesity** (WtHR) (No/Yes)	2.02 (1.78–2.31)	*p* = 0.0021
**Abdominal obesity** (WC) (No/Yes)	1.98 (1.75–2.24)	*p* = 0.0038
**Birth weight** (Low & normal/High)	1.88 (1.37–2.39)	*p* = 0.1563
**Physical activity** ^2^ (Low/Medium & High)	1.57 (1.21–1.98)	*p* = 0.0175
**Quality of life** ^3^ (Below/Over mean value)	2.08 (1.85–2.31)	*p* = 0.0086
**Exclusive breastfeeding** (No/Yes)	2.19 (1.98–2.35)	*p* = 0.0056
**Asthma** (No/Yes)	1.69 (1.34–2.03)	*p* = 0.0182
**Diabetes type 1** (No/Yes)	1.48 (1.13–1.86)	*p* = 0.0289

* Odds Ratio: OR ** CI: Confidence Interval ^1^ Mediterranean diet (MD) adherence was assessed by the KIDMED (Mediterranean Diet Quality Index for children and adolescents) questionnaire [55]. ^2^ Physical activity was assessed by the Preschool-age Children’s Physical Activity Questionnaire (Pre-PAQ) [53]. ^3^ Quality of life was assessed by the Pediatric Quality of Life Inventory (PedsQL) questionnaire [54].

## Data Availability

The data of the study are available upon request to the corresponding author.

## Supplementary Materials

The following supporting information can be downloaded at: https://www.mdpi.com/article/10.3390/epidemiologia5010002/s1. Supplementary Files S1–S3.
