# Supplementary material for: Childhood Mediterranean Diet Adherence Is Associated with Lower Prevalence of Childhood Obesity, Specific Sociodemographic, and Lifestyle Factors: A Cross-Sectional Study in Pre-School Children
Source: Epidemiologia (Basel). 2023 Dec 23;5(1):11–28. doi: 10.3390/epidemiologia5010002 (PMC10801514; doi:10.3390/epidemiologia5010002)
Supplement: Supplementary file 1 [file epidemiologia-05-00002-s001.zip › Suplementary File S1/Pre-PAQ.Part.B.pdf]

Q37 Which of the following activities did your child do LAST WEEKEND?

(record “0” for any activities that your child did not do)

			Saturday			Sunday
	Did your child do this activity		Total time spent in activity?	Did your child do this activity?		Total time spent in activity
	Yes	No	Hours/Minutes	Yes	No	Hours/Minutes
Sat or lay still watching TV	☐	☐	☐hrs ☐☐ mins	☐	☐	☐hrs ☐☐ mins
Sat or lay still watching a DVD or a video	☐	☐	☐hrs ☐☐ mins	☐	☐	☐hrs ☐☐ mins
Sat or lay still (e.g., looking at books or listening to stories)	☐	☐	☐hrs ☐☐ mins	☐	☐	☐hrs ☐☐ mins
Played computer or electronic games	☐	☐	☐hrs ☐☐ mins	☐	☐	☐hrs ☐☐ mins
Was **stationary** but moving a part of the body such as swimming or swaying trunk (e.g., standing and swaying to a song) or moving arm or leg (e.g., sitting doing puzzles or craft, digging in a sandpit or standing and kicking or throwing a ball, doing movements to a song)	☐	☐	☐hrs ☐☐ mins	☐	☐	☐hrs ☐☐ mins
Walked at a **leisure or moderate pace** (from any reason – not just when going a walk)	☐	☐	☐hrs ☐☐ mins	☐	☐	☐hrs ☐☐ mins
Walked at a **fast pace**	☐	☐	☐hrs ☐☐ mins	☐	☐	☐hrs ☐☐ mins
Walked up steel slopes	☐	☐	☐hrs ☐☐ mins	☐	☐	☐hrs ☐☐ mins
Ran or jogged **slowly**	☐	☐	☐hrs ☐☐ mins	☐	☐	☐hrs ☐☐ mins
Ran or jogged **quickly**	☐	☐	☐hrs ☐☐ mins	☐	☐	☐hrs ☐☐ mins
Rough and tumble play with **moderate effort**	☐	☐	☐hrs ☐☐ mins	☐	☐	☐hrs ☐☐ mins
Rough and tumble play with **hard effort**	☐	☐	☐hrs ☐☐ mins	☐	☐	☐hrs ☐☐ mins

ALL INFORMATION WILL BE KEPT STRICTLY CONFIDENTIAL

	Saturday			Sunday		
	Did your child do this activity		Total time spent in activity?	Did your child do this activity?		Total time spent in activity
	**Yes**	**No**	**Hours/Minutes**	**Yes**	**No**	**Hours/Minutes**
Hopped, jumped, skipped or marched at an **easy pace**	☐	☐	☐hrs ☐☐ mins	☐	☐	☐hrs ☐☐ mins
Hopped, jumped, skipped or marched with a **moderate speed or effort**	☐	☐	☐hrs ☐☐ mins	☐	☐	☐hrs ☐☐ mins
Hopped, jumped, skipped or marched with **fast speed or hard effort**	☐	☐	☐hrs ☐☐ mins	☐	☐	☐hrs ☐☐ mins
Danced or did movement and music activities (moving around)	☐	☐	☐hrs ☐☐ mins	☐	☐	☐hrs ☐☐ mins
Climbed (e.g., on play equipment, in a tree etc.)	☐	☐	☐hrs ☐☐ mins	☐	☐	☐hrs ☐☐ mins
Used swing (moving self. Not being pushed by another person)	☐	☐	☐hrs ☐☐ mins	☐	☐	☐hrs ☐☐ mins
Rode a tricycle, bike or scooter etc. at an **easy pace or slow speed**	☐	☐	☐hrs ☐☐ mins	☐	☐	☐hrs ☐☐ mins
Rode a tricycle, bike or scooter etc. at a **moderate pace or medium speed**	☐	☐	☐hrs ☐☐ mins	☐	☐	☐hrs ☐☐ mins
Swam by self (± floatation devices)	☐	☐	☐hrs ☐☐ mins	☐	☐	☐hrs ☐☐ mins
Swam with support of an adult	☐	☐	☐hrs ☐☐ mins	☐	☐	☐hrs ☐☐ mins
Other (please state)	☐	☐	☐hrs ☐☐ mins	☐	☐	☐hrs ☐☐ mins
Other (please state)	☐	☐	☐hrs ☐☐ mins	☐	☐	☐hrs ☐☐ mins

Thank you for completing this questionnaire

ALL INFORMATION WILL BE KEPT STRICTLY CONFIDENTIAL

ALL INFORMATION WILL BE KEPT STRICTLY CONFIDENTIAL
