# Supplementary material for: Childhood Mediterranean Diet Adherence Is Associated with Lower Prevalence of Childhood Obesity, Specific Sociodemographic, and Lifestyle Factors: A Cross-Sectional Study in Pre-School Children
Source: Epidemiologia (Basel). 2023 Dec 23;5(1):11–28. doi: 10.3390/epidemiologia5010002 (PMC10801514; doi:10.3390/epidemiologia5010002)
Supplement: Supplementary file 1 [file epidemiologia-05-00002-s001.zip › Supplementary File S2.pdf]

Date:________________________

ID#_________________________

PedsQL

Paediatric Quality of life Inventory

Version 4.0

PARENT REPORT for **TODDLERS** (ages 2-4)

DIRECTIONS

On the following page is a list of things that might be a problem for **your child**.
Please tell us how much of a problem each one has been for your child during the **PAST MONTH** by circling:

0 if it is **never** a problem
1 if it is **almost never** a problem
2 if it is **sometimes** a problem
3 if it is **often** a problem
4 if it is **almost always** a problem

There are no right or wrong answers.
If you do not understand a question, please ask for help.

*In the **PAST MONTH**, how much of a **problem** has your child had with …*

**PHYSICAL FUNCTIONING (problems with …)**	**Never**	**Almost Never**	**Some-times**	**Often**	**Almost Always**
1. Walking	0	1	2	3	4
2. Running	0	1	2	3	4
3. Participating in active play and exercise	0	1	2	3	4
4. Lifting heavy things	0	1	2	3	4
5. Bathing	0	1	2	3	4
6. Helping to pick up his or her toys	0	1	2	3	4
7. Having aches or pains	0	1	2	3	4
8. Feeling tired	0	1	2	3	4

**EMOTIONAL FUNCTIONING (problems with …)**	**Never**	**Almost Never**	**Some-times**	**Often**	**Almost Always**
1. Feeling afraid or scared	0	1	2	3	4
2. Feeling sad	0	1	2	3	4
3. Feeling angry	0	1	2	3	4
4. Having trouble sleeping	0	1	2	3	4
5. Worrying	0	1	2	3	4

**SOCIAL FUNCTIONING (problems with …)**	**Never**	**Almost Never**	**Some-times**	**Often**	**Almost Always**
1. Playing with other children	0	1	2	3	4
2. Other children not wanting to play with him or her	0	1	2	3	4
3. Getting teased by other children	0	1	2	3	4
4. Not able to do things that other children his or her age can do	0	1	2	3	4
5. Keeping up when playing with other children	0	1	2	3	4

Please complete this section if your child attends nursery or day care

**NURSERY/DAY CARE FUNCTIONING (problems with …)**	**Never**	**Almost Never**	**Some-times**	**Often**	**Almost Always**
1. Doing the same nursery/day care activities as peers	0	1	2	3	4
2. Missing nursery/day care because of not feeling well	0	1	2	3	4
3. Missing nursery/day care to go to the doctor or hospital	0	1	2	3	4
