# Supplementary material for: Childhood Mediterranean Diet Adherence Is Associated with Lower Prevalence of Childhood Obesity, Specific Sociodemographic, and Lifestyle Factors: A Cross-Sectional Study in Pre-School Children
Source: Epidemiologia (Basel). 2023 Dec 23;5(1):11–28. doi: 10.3390/epidemiologia5010002 (PMC10801514; doi:10.3390/epidemiologia5010002)
Supplement: Supplementary file 1 [file epidemiologia-05-00002-s001.zip › Supplementary File S3 .pdf]

## KIDMED QUESTIONNAIRE

| | | |
|---|---|---|
| **1.** Takes a fruit or fruit juice every day? | Yes □ No □ | |
| **2.** Has a second fruit every day? | Yes □ No □ | |
| **3.** Has fresh or cooked vegetables regularly once a day? | Yes □ No □ | |
| **4.** Has fresh or cooked vegetables more than once a day? | Yes □ No □ | |
| **5.** Consumes fish regularly (at least 2-3/week)? | Yes □ No □ | |
| **6.** Goes > 1 / week to a fast-food restaurant (hamburger)? | Yes □ No □ | |
| **7.** Likes pulses and eats them > 1 / week | Yes □ No □ | |
| **8.** Consumes pasta or rice almost every day (5 or more per week)? | Yes □ No □ | |
| **9.** Has cereals or grains (bread, etc) for breakfast? | Yes □ No □ | |
| **10.** Consumes nuts regularly (at least 2-3/week)? | Yes □ No □ | |
| **11.** Uses olive oil at home? | Yes □ No □ | |
| **12.** Skips breakfasts? | Yes □ No □ | |
| **13.** Has a dairy product for breakfast (yogurt, milk, etc)? | Yes □ No □ | |
| **14.** Has commercially baked goods or pastries for breakfast? | Yes □ No □ | |
| **15.** Takes two yogurts and/or some cheese (40g) daily? | Yes □ No □ | |
| **16.** Takes sweets and candy several times every day? | Yes □ No □ | |
| | **KMS:** | |
